# 

**Published:** 1971-01

**Authors:** 


The
Bristol Medico-Chirurgical Journal
(Incorporating the Medical Journal of the South-West)
Established 1883

### BRISTOL MEDICO-CHIRURGICAL JOURNAL

**Vol. 86**
**1971**

Editor: N. J. Brown, M.B., F.R.C.P., F.R.C.Path.

Published Quarterly

Annual Subscription 16/- (80p) Post Free

### BRISTOL MEDICO-CHIRURGICAL SOCIETY

**President 1970-71 : Dr. Beryl Corner**

**President-elect : Dr. C. C. Morgans**

**Honorary Secretary : Dr. I. S. Bailey, 7 Percival Road, Bristol 8.**

**Honorary Treasurer : Dr. Frank Ross, Bristol Royal Infirmary, Bristol 2.**

The Society was founded in 1874. In 1883 publication of the Journal began and has continued quarterly ever since then. During the years 1949 to 1962 it appeared under the title of "The Medical Journal of the South West".

### THE BRISTOL MEDICO-CHIRURGICAL JOURNAL

### Editorial Committee

| | |
|---|---|
| Editor | N. J. Brown, Southmead Hospital, Bristol. |
| Assistant Editor | Mr. J. B. M. Roberts. |
| Members | Dr. M. B. Lennard.<br>Professor R. C. Wofinden.<br>Dr. D. W. Wright.<br>The Hon. Treasurer and Hon. Secretary of the Society. |
| Business Manager | Dr. P. J. F. Baskett, Frenchay Hospital, Bristol. |
| Secretary | Mr. B. P. Jones, The Library, Medical School, University Walk, Bristol BS8 1TD. |

### NOTICE TO CONTRIBUTORS

The Journal is especially concerned to provide a place for the recording of medical thought, interests, and practice in and around Bristol. It therefore welcomes articles that, as well as being of immediate interest to members, will document the local and contemporary medical scene for our successors.

Original articles are invited provided that they have not been published elsewhere. References in the text should be by author's name, with year of publication in parenthesis; they should be listed at the end in alphabetical order, the name of each journal or book being given in full — not abbreviated — and the title of the article added. Illustrations should be supplied ready for reproduction. Line drawings should be in Indian ink on firm white paper or board. The author will be informed if it is found necessary to ask him to pay for the making of blocks.

The following contributions will be especially welcome: notes of forthcoming events, meetings held, degrees conferred, staff appointments, honours and public appointments and other local news.

Twenty-five reprints of his article will be supplied free to each contributor. Quotations for larger quantities will be sent out with proofs and must be ordered when the corrected proofs are returned.

### The Medical Library

In 1890 the Bristol Medico-Chirurgical Society founded a medical library which in 1892 was combined with that of the Bristol Medical School. This became the University of Bristol Medical Library in 1925 and an agreement in that year confirmed the privilege of Members of the Society to use the University Medical Library.

### A Selection of Recent Additions to the Medical Library

ADAMS, C. P.: Design and construction of removable orthodontic appliances. Ed. 4 (Wright). Presented by the publishers.

ANDREWS, R. D.: Viral interference and interferon. (Heinemann)

BARLOVATZ, A.: Principles of rational medicine. (Wright). Presented by the publishers.

BARTELS, H.: Prenatal respiration. (North Holland)

BLACK, P.: Drugs and the brain. (Johns Hopkins)

BOTSFORD, T. W. & WILSON, R. E.: The acute abdomen. (Saunders)

BOYARSKI, S. ed.: The neurogenic bladder. (Livingstone)

BOYD, J. D. & HAMILTON, W. J.: The human placenta. (Heffer)

BRAMBELL, F. W. R.: Transmission of passive immunity from mother to young. (North Holland)

BRAUN, A. C.: The cancer problem. (Columbia Univ. Pr.)

BROWNE, J. C M. & DIXON, G.: Antenatal care. Ed. 10, (Churchill)

BURSTON, G. R.: Self-poisoning. (Lloyd-Luke)

CARTER, C. O.: An ABC of medical genetics. (Lancet)

CARTER, R. L. & PENMAN, H. G.: Infectious mononucleosis. (Blackwell)

DAGLEY, S. & NICHOLSON, D. E.: Introduction to metabolic pathways. (Blackwell)

DICK, D. A. T.: Cell water. (Butterworths)

EASTHAM, R. D.: Clinical haemotology. Ed. 3 (Wright). Presented by the publishers

EVERSON, T. C. & COLE, W. H.: Cancer of the digestive tract. (Butterworths)

FORD, A. B. et al.: The doctor's perspective. (Case Western Reserve Univ. Pr.)

GARDNER, A. F.: Differential oral diagnosis in systemic disease. (Wright) Presented by the publishers

GASTAUT, H. et al.: The physiopathogenesis of the epilepsies. (Thomas).

GOLD, E. R. & PEACOCK, D. B.: Basic immunology. (Wright) Presented by the publishers

GOOCH, A. S. et al.: Clues to diagnosis in congenital heart disease. (Davis)

GOODLAND, N. L.: Coronary care. (Wright) Presented by the publishers

GROSS, L.: Oncogenic viruses. (Pergamon)

HARGREAVES, T.: Liver and bile metabolism. (Appleton)

HEARD, B. E.: Pathology of chronic bronchitis and emphysema. (Churchill)

HOOPER, R.: Patterns of acute head injury. (Arnold)

JACO, E. G.: Patients, physicians and illness. (Free Pr.)

JONES, P. G. ed.: Clinical paediatric surgery. (Wright). Presented by the publishers

KOSA, J. et al. eds.: Poverty and health. (Harvard Univ. Pr.)

MACCONAILL, M. A. & BASMAJIAN, J. V.: Muscles and movement. (Livingstone)

McGREGOR, R. M.: The work of a family doctor, (Livingstone)

McKERNS, K. W. ed.: The gonads. (Appleton)

McMINN, R. M. H.: Tissue repair. (Academic Pr.)

MEEK, E. S.: Antitumour and antiviral substances of natural origin. (Springer)

MILLICHAP, J. G.: Febrile convulsions. (Macmillan)

MORRIS, S. C.: A complete handbook for professional ambulance personnel. (Wright) Presented by the publishers

MORSON, B. C.: Diseases of the colon, rectum and anus. (Heinemann)

NOSSAL, G. J. V.: Antibodies and immunity. (Basic Books)

PEARSON, L.: Death and dying. (Case Western Reserve Univ. Pr.)

PLOG, S. C. & EDGERTON, R. B. eds.: Changing perspectives in mental illness. (Holt)

RHODES, P.: Reproductive physiology for medical students. (Churchill)

RICH, M. A.: Experimental leukaemia. (North Holland)

ROBERTS, D. H. & SOWRAY, J. H.: Local analgesia in dentistry. (Wright) Presented by the publishers

SAMPSON, G. R.: Public supplies work. (Wright). Presented by the publishers

SARMA, K. P.: Tumours of the urinary bladder. (Butterworths)

SAWIN, C. T.: The hormones. (Churchill)

SCOTT, W. R. & VOLKART, E. H.: Medical care (Wiley)

SHUSTER, S. & MARKS, J.: Systemic effects of skin diseases. (Heinemann)

STACEY, M. et al.: Hospitals, children and their families. (Routledge)

STEVENS, P. J.: Fatal civil aircraft accidents. (Wright) Presented by the publishers

STEVENSON, A. C. & DAVISON, B. C. C.: Genetic counselling. (Heinemann)

TUFT, L. & MUELLER, H. L.: Allergy in children. (Saunders)

UPTON, A. C.: Radiation injury. (Chicago Univ. Pr.)

WALFORD, R. L.: Immunologic theory of ageing. (Munksgaard).

WALLACE, B.: Genetic load. (Prentice-Hall)

WARIN, J. F. & IRONSIDE, A. G.: Lecture notes on the infectious diseases. (Blackwell)

WESTPHAL, O. et al.: Current problems in immunology. (Springer)

WITTEN, D. M.: The breast. (Wiley)

WOOD, C.: Birth control now and tomorrow. (Peter Davies)

WOODFORD, P.: Scientific writing for graduate students. (Macmillan)

### Bristol Medico-Chirurgical Society

The **Annual General Meeting** was held in the Medical School on 14th October. Dr. N. J. Brown showed pathological specimens, Dr. G. R. Airth demonstrated skiagrams and Mr. B. P. Jones arranged a display of medical books. The retiring president, Dr. F. J. W. Lewis, introduced his successor, Dr. Beryl Corner, who then addressed the Society on the subject of Beethoven. The text of this novel and fascinating study—but not, unfortunately, its musical illustrations—will be published in a later number of the Journal. The following officers were elected—President-elect, Dr. C. C. Morgans; Hon. Secretary, Dr. Ian Bailey; Hon. Treasurer, Dr. F. Ross; Hon. Medical Librarian, Dr. G. E. F. Sutton; new members of committee, Dr. A. Macleod, Dr. S. J. Silvey and Dr. J. Telling; members to serve on University Library Medical Sub-committee, Prof. A. E. Read, Dr. M. Lennard.

The second meeting of the session was held on 11th November. Dr. A. C. Hunt showed pathological specimens and Mr. B. P. Jones arranged a display of medical books. The guest speaker, **Mr. Justice Lawton,** addressed the Society on "Violence in Britain Today."

The **Annual Clinical Meeting** of the Society was held in the Bristol Royal Hospital for Sick Children on December 9th.

### South-West Orthopaedic Club

A combined meeting of the South-West Orthopaedic Club and the St. Mary's Hospital Orthopaedic Club was held at the Princess Elizabeth Orthopaedic Hospital, Exeter on 16th May, 1970.

**Mr. R. S. M. Ling** (Exeter) reported ten cases of deep infection in 268 operations for the insertion of **McKee-Farrar hip prostheses,** an incidence of 3.7 per cent; in 4 per cent the prostheses were removed. In eight of the ten hips, infection occurred late, but in two of these there were grounds for believing that the hips were not infected at operation but directly or indirectly from the blood stream at a later date. In this type of situation an aggressive approach to the infection was justified, with drainage of the hip followed by irrigation with antibiotics and detergent.

**Mr. J. E. Woodyard (Exeter)** presented the results of a study of 153 patients who had had **prosthetic replacement for sub-capital fractures of the neck of the femur.** One group of patients with Austin Moore prostheses was compared with another with Thompson prostheses fixed with bone cement. Seven out of twenty-five patients with Austin Moore prostheses had no deterioration of function whereas sixteen of the twenty-six patients with cemented Thompson prostheses had not deteriorated. It was considered that the use of the Austin Moore prosthesis should be discontinued in favour of the Thompson prosthesis fixed with bone cement.

**Mr. F. C. Durbin (Exeter)** directed attention to the classification of **congenital angular deformities of the tibia** by Heyman and Herndon in 1949 into anterior and posterior groups. The latter was less common but tended to correct itself spontaneously in the course of a few years. He reported thirteen cases of bowing seen at Exeter in the last thirty years—twelve anterior and one posterior. There was one case of infantile pseudarthosis in the anterior group. Three cases were presented, one with congenital anterior angular deformity of the right tibia, both femora and the humeri. The patient had blue sclerotics indicating a congenital defect of the mesenchyme. The deformity corrected spontaneously. Two other cases were presented of closed correction of the deformity by manipulative fracture separation of the lower tibial epiphysis, with successful results—one followed up for twenty years.

**Prof. J. Mulier (Louvain)** showed a film of an operation which he had found of value in both the primary treatment of **subluxation of the patella** and in failed cases following other forms of surgical treatment. The operation involved the loop of gracilis and semimembranosus around the outer side of the patella to potentiate the action of vastus medialis.

**Mr. A. L. Eyre-Brook (Bristol)** gave an account of his experience with Pemberton's pelvic osteotomy in **congenital dislocation of the hip.** He had found it a valuable procedure where there was a failure of acetabular development. An illustrated description of the operative technique was given.

**Mr. G. Blundell Jones (Exeter)** spoke of the results and the experience gained from thirty-six pelvic osteotomies of the Salter type performed for **congenital dislocation of the hip.** In fifteen it was the primary treatment and in twenty-one was done following failure of either conservative or operative treatment. He advised opening the hip in order to remove any obstruction to deep seating of the head in the acetabulum, measuring the degree of anteversion and seeing the extent of the acetabular roof correction obtained by the ostectomy. If, at operation, cover of the femoral head with the leg in the neutral position appeared to be inadequate, he thought that it might be advisable to proceed immediately to a femoral ostectomy.

**Mr. C. C. Jeffery and Dr. G. Stewart-Smith (Exeter)** gave an account of the clinical and histological features of three cases of **hamartoma of the hand.**

**Mr. N. Capener (Exeter)** spoke of the causation of **accidents,** of their complexity and of the therapeutic team work necessary in the handling of the injured.

**Mr. R. Snook (Bath)** described the **Accident Flying Squad** presently operating in Bath, taking medical staff and equipment to the scene of accidents, especially those involving trapped casualties. Photographs were shown of the medical and rescue equipment needed. It was apparent that this service had saved lives and eased suffering.

**Surgeon Commander J. Bertram (Royal Navy)** reviewed eighty-four **stress fractures** occurring in servicemen. On clinical and radiological grounds two types could be defined—distraction and compression. The different patterns of fracture occurring with different activities were emphasised and the mechanical factors involved were discussed. It was felt that the stress of muscle action was more important than the stress of weight-bearing in the causation of stress fractures because of certain clinical observations and mechanical factors.

**Dr. R. F. Payne (Bridgwater)** reported the results of the first hundred **hip fractures** treated in the North Devon Accident Service with a mean follow-up of two years. There was an overall mortality of 10 per cent within one month and of 30 per cent within one year. There were 47 trochanteric fractures all treated by McLaughlin nail plating and early weight bearing with a 35 per cent mortality in the first year. In the survivors there were no failures of union and no residual hip disability. There were 53 intracapsular fractures. Grade 1 and 2 fractures treated by low angle nailing had uniformly good results. Grade 3 fractures were treated by primary nailing but over a third required a secondary prosthesis.

**Mr. P. J. McGrath (Bristol)** reviewed 49 cases of **giant cell tumour of bone** from the Bristol Bone Tumour Registry, and concluded that careful curettage and graft was the best form of primary treatment.

